# Personal Notes

**Published:** 1890-04

**Authors:** 


### PERSONAL NOTES.

Dr. K. P. Moore, the present Secretary of the Medical Association of Georgia, has exceeded himself in the general get-up of the programme and announcement for the approaching meeting of the Association at Brunswick. For good taste and neatness we think this bit of his work can hardly be excelled.

We all know that Dr. Moore did not accept the Secretary's office for the honors attached to it. He had already enjoyed all the honors that the Association could bestow, from the Presidency down; and in assuming the hard duties of Secretary, he was prompted only by his ardent love and attachment for the Association.

The last volume of Transactions was a model of neatness and good taste, and reflects credit not only on the Secretary but upon the Association. That the Association may for many years have Dr. Moore as its Secretary is the wish, I have no doubt, of all its members. We give place elsewhere to the programme and invitation.

Dr. C. D. Roy, junior assistant of the Charity Hospital of New York, is on a visit to Atlanta. Dr. Roy is a correspondent of the RECORD, and we extend to him the compliments of the season. We wish him great success in his new field of labor. He will stay in New York one year longer, when he will return to our city, and go in with his esteemed father, Dr. G. G. Roy.

Dr. George Ross, Chief Surgeon of the Richmond & Danville Railroad, passed through the city on the 12th instant, en route to Mexico.

We had a very pleasant call from Mr. F. A. Davis, of Philadelphia. Mr. Davis is running one of the largest publishing houses in the United States for medical works. He spent several days in our city, and we had the pleasure of seeing him, both socially and in business ways. We would like to get all such gentlemen as he to move South. He has just been to Florida, looking after his landed interest, and has established a branch house in our city. As soon as he sees the advantages that Atlanta offers over Philadelphia he will move here immediately. We hope him great success with his branch house, under the management of Dr. Head. He could not have made a better selection. We advise the medical profession of our State, if they wish to buy medical books, to send their orders to Dr. Head, of this city.

